# Improved succinic acid production through the reconstruction of methanol dissimilation in *Escherichia coli*

**DOI:** 10.1186/s40643-022-00547-x

**Published:** 2022-05-31

**Authors:** Feng Guo, Min Wu, Shangjie Zhang, Yifan Feng, Yujia Jiang, Wankui Jiang, Fengxue Xin, Wenming Zhang, Min Jiang

**Affiliations:** 1 State Key Laboratory of Materials-Oriented Chemical Engineering, College of Biotechnology and Pharmaceutical Engineering, Nanjing Tech University, Puzhu South Road 30#, Nanjing, 211800 People’s Republic of China; 2 Jiangsu National Synergetic Innovation Center for Advanced Materials (SICAM), Nanjing Tech University, Nanjing, 211800 People’s Republic of China

**Keywords:** Methanol dissimilation, C1-substrates utilization, Succinic acid fermentation, CO_2_ fixation, Synthetic biology

## Abstract

Synthetic biology has boosted the rapid development on using non-methylotrophy as chassis for value added chemicals production from one-carbon feedstocks, such as methanol and formic acid. The one-carbon dissimilation pathway can provide more NADH than monosaccharides including glucose, which is conducive for reductive chemicals production, such as succinic acid. In this study, the one-carbon dissimilation pathway was introduced in *E. coli* Suc260 to enhance the succinic acid production capability. Through the rational construction of methanol dissimilation pathway, the succinic acid yield was increased from 0.91 to 0.95 g/g with methanol and sodium formate as auxiliary substrates in anaerobic fed-batch fermentation. Furthermore, the metabolic flux of by-product pyruvate was redirected to succinic acid together with the CO_2_ fixation. Finally, through the immobilization on a specially designed glycosylated membrane, *E. coli* cells are more resistant to adverse environments, and the final yield of succinic acid was improved to 0.98 g/g. This study proved the feasibility of endowing producers with methanol dissimilation pathway to enhance the production of reductive metabolites.

**Graphical Abstract:**

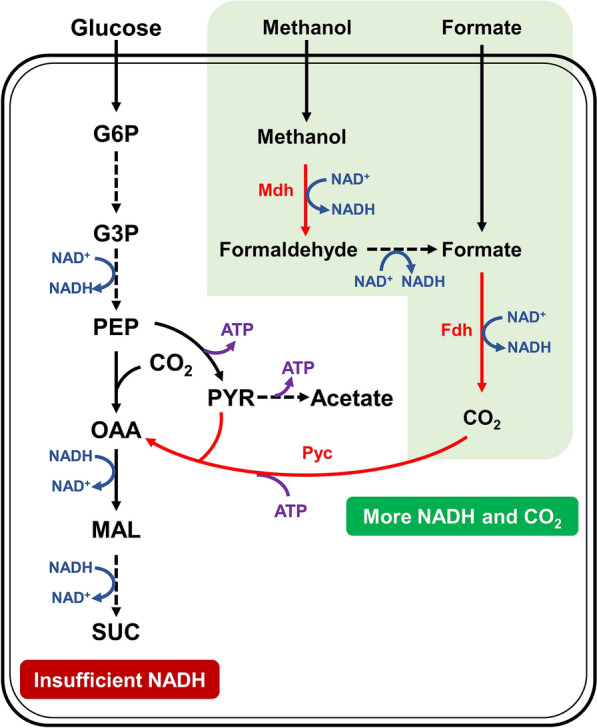

**Supplementary Information:**

The online version contains supplementary material available at 10.1186/s40643-022-00547-x.

## Introduction

The depletion of fossil fuels and concerns on environmental issues have aroused the interest of industrial bio-manufacturing, which can synthesize various bulk and fine chemicals from renewable resources (Isikgor and Becer 2015; Zhang et al. 2018a). As one of the most important platform chemicals, succinic acid has been identified as one of the top 12 building block chemicals by the US Department of Energy, which has been widely used as surfactant, ion chelator, acidulate agent and additive in metal, food, and pharmaceutical industries (Cheng et al. 2012; Minh et al. 2010; Werpy et al. 2006; Zeikus et al. 1999). Furthermore, it is also a precursor for the synthesis of biodegradable polymers, such as polybutylene succinate (PBS) and polyamides (Nylon x,4) (Cheng et al. 2012).

In recent years, the bio-production of succinic acid from renewable resources through the microbial fermentation has gained great attentions (Bradfield et al. 2015; Chen et al. 2016; Dai et al. 2020; Jian et al. 2016; Li et al. 2018). Being an intermediate of tricarboxylic acid (TCA) cycle, succinic acid is also a reducing end-product of some bacterial strains under anaerobic conditions, such as *Escherichia coli* (Wang et al. 2011; Zhang et al. 2018b), *Actinobacillus succinogenes* (Guarnieri et al. 2017; McKinlay and Vieille 2008) and *Mannheimia succiniciproducens* (Lee et al. 2006, 2016), etc. To reduce the production cost, some low-cost feedstocks have been explored to produce succinic acid through microbial fermentation, including corn stalk (Chen et al. 2010a; Yu et al. 2010), sugarcane bagasse (Chen et al. 2016), sake lees (Chen et al. 2010b), coffee husk (Dessie et al. 2018) and so on. Notably, one mole CO_2_ is fixed for the production of one mole succinic acid during the anaerobic fermentation (Dai et al. 2020). In this case, the maximum theoretical yield for succinic acid is 2 mol/mol of glucose (Dai et al. 2020). However, two moles NADH are needed for the production of one mole succinic acid, leading to lower factual yield due to the shortage of NADH within the fermentative microbes.

One-carbon (C1) compounds such as methanol, formic acid and methane have emerged as alternative feedstocks for microbial fermentation owning to their low cost, high abundance and even higher content of reducing equivalents (Liu et al. 2018; Sehested 2019; Wang et al. 2017). Compared with syngas, these C1 compounds are highly soluble, which have less mass transfer barrier and are easily stored and transported (Jiang et al. 2021a). For example, formic acid can be easily obtained from one carbon gases through the electrochemical reduction and hydrogenation (Bang and Lee 2018). In addition, various studies have reported that formic acid can be used as a secondary carbon source for the production of value-added products (Ahn et al. 2017). Another typical C1 compound is methanol, which is highly reduced and can provide more reducing equivalent than mono-sugars. This will benefit for the production of reductive chemicals including alcohols and organic acids (Sehested 2019). In general, methanol was mainly used by native methylotrophs to produce single-cell proteins and various amino acids (Irla et al. 2016; Mokhtari‐Hosseini et al. 2009; Sonntag et al. 2014; Wegner 1990). However, the lack of efficient genetic tools for methylotrophs hinders their further application. Alternatively, metabolic construction of synthetic methylotrophs arise great interests through the introduction of the methanol assimilation module into some model organisms, including *E. coli*, *Corynebacterium glutamicum* and *Saccharomyces cerevisiae* (Muller et al. 2015; Naerdal et al. 2015; Wang et al. 2017; Whitaker et al. 2017). In the last few years, several groups have made significant progress towards genetic modification of these non-native methylotrophs to achieve the assimilation of methanol into various chemicals production, such as pyruvic acid, ethanol, naringenin, etc. (Dai et al. 2017; Whitaker et al. 2017).

**Fig. 1:**
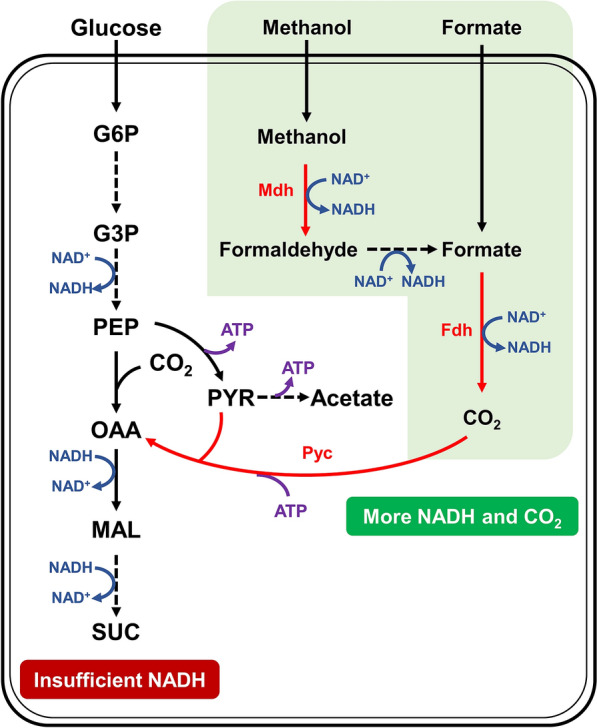
Diagram showing the metabolic pathway of succinic acid of engineered *E. coli* under anaerobic condition. The dissimilation metabolic pathway of methanol and formate and a new pathway of fixing CO2 were introduced. *G6P* glucose-6-phosphate, *G3P* 3-phosphate-glyceraldehyde, *PEP* phosphoenolpyruvic acid, *OAA* oxaloacetate, *PYC* pyruvate, *Mdh* methanol dehydrogenase, *Fdh* formate dehydrogenase, *Pyc* pyruvate carboxylase

In our previous study, we have successfully genetically constructed a methylotrophic and succinic acid producing *E. coli* through the introduction of methanol assimilation module harboring NAD^+^-dependent methanol dehydrogenase (Mdh) from *Bacillus methanolicus* and ribulose monophosphate pathway from *B. methanolicus* or *B. subtilis* (Zhang et al. 2018a). To further improve its succinic acid production capability, the dissimilation pathways of methanol and formate will be introduced to generate more NADH (Fig. 1). In addition, its CO_2_ fixation capability will be enhanced by introducing pyruvate carboxylase from *Lactococcus lactis*. Finally, a glycosylated membrane will be applied to immobilize *E. coli* cells and improve its resistance towards these toxic C1-substrates. Overall, this study will provide a new strategy for one-carbon material bioconversion using methanol and formate as the auxiliary substrates for chemicals production.

## Materials and methods

### Strains and plasmids

All strains and plasmids used in this study are listed in Table 1. Primers used in this study are listed in Additional file 1: Table S1. Methanol or formate dissimilation genes were expressed and characterized using the pTrc99A vector. *E. coli* DH5α was selected to propagate all plasmids, while *E. coli* Suc260 (a derivate of *E. coli* BER208, China Center for Type Culture Collection, CCTCC NO: M 2012351) was used as the host for succinic acid production.

**Table 1:** Strains and plasmids used in this study

Name	Strains	Genotype	Source
DH5α	*E. coli* DH5α	*endA1*, *hsdR17* (*rk−*, *mk*+), *phoA*, *supE44λ−*, *thi-1*, *gyrA96*, *relA1*	Invitrogen
Suc260	*E. coli* Suc260	Adaptive mutant of *E. coli* BER208	Zhang et al. (2018a)
G0	*E. coli* Suc360	Suc260/pTrc99A	Zhang et al. (2018a)
G1	*E. coli* Suc361	Suc260/pTrc99A/*mdh2*	This study
G2	*E. coli* Suc362	Suc260/pTrc99A/*mdh3*	This study
G3	*E. coli* Suc363	Suc260/pTrc99A/*mdh2*/*fdhABCD*	This study
G4	*E. coli* Suc364	Suc260/pTrc99A/*mdh2/fdh1*	This study
G5	*E. coli* Suc365	Suc260/pTrc99A/*mdh2*/*fdh1/pyc*	This study
Plasmid			
pTrc99A		*AmpR*, *Ptrc*, pBR322_origin	Invitrogen
pUC18-*mdh2*		*AmpR*, *Plac*, pBR322_origin	Genscript
pUC18-*mdh3*		*AmpR*, *Plac*, pBR322_origin	Genscript
pTrc99A-*mdh2*		*AmpR*, *Ptrc*, *B. methanolicus mdhB*	This study
pTrc99A-*mdh3*		*AmpR*, *Ptrc*, *B. stearothermophilus mdh*	This study
pTrc99A-*fdhABCD*		*AmpR*, *Ptrc*, *M. extorquens fdhABCD*	This study
pTrc99A-*mdh2-fdh1*		*AmpR*, *Ptrc*, *B. methanolicus mdh*, *C. boidinii fdh*	This study
pTrc99A-*mdh2-fdhABCD*		*AmpR*, *Ptrc*, *B. methanolicus mdh*, *M. extorquens fdhABCD*	This study
plgz920-*pyc*		*AmpR*, *Ppck*, 130Z_origin	(Liang et al. 2012)
pTrc99A-*pyc*		*AmpR*, *Ptrc*, *Lactococcus lactis pyc*	Genscript
pTrc99A-*mdh1-fdh1-pyc*		*AmpR*, *Ptrc*, *B. methanolicus mdh*, *C. boidinii fdh*, *Lactococcus lactis pyc*	This study

The genes *mdh2* originating from *B. methanolicus* MGA3, *mdh3* originating from *B. stearothermophilus* 2334 and *fdh1* originating from *Candida boidinii* were synthesized and codon optimized for expression in *E. coli* by Genscript. The genes *fdhABCD* and *pyc* were prepared via PCR amplification from the genomic DNA of *Methylobacterium extorquens* and *Lactococcus lactis*, respectively. For construction of pTrc99A-*mdh2* and pTrc99A-*mdh3*, the genes *mdh2* and *mdh3* were integrated into the vector pTrc99A digested with EcoRI by One Step Cloning Kit (Vazyme, C112-02), respectively. The other respective fragments such as genes *fdh1* and *fdhABCD* were further inserted into pTrc99A-*mdh2* digested with XbaI using Gibson assembly, respectively. The gene *pyc* was then inserted into pTrc99A-*mdh2-fdh1* digested with HindIII using Gibson assembly. The resulting expression vector was called pTrc99A-*mdh2*, pTrc99A-*mdh3*, pTrc99A-*mdh2-fdh1*, pTrc99A-*mdh2-fdhABCD*, and pTrc99A-*mdh2-fdh1-pyc*. Finally, those plasmids were transformed into *E. coli* Suc260 to construct the recombinant strains named as G1, G2, G3, G4 and G5.

### Media and growth conditions

Luria–Bertani (LB) or M9 medium was used for strain construction and cultivation. Fermentation was carried out in chemically defined (CD) medium (3 g/L citric acid, 4 g/L Na_2_HPO_4_·12H_2_O, 8 g/L KH_2_PO_4_, 8 g/L (NH_4_)_2_HPO_4_, 0.2 g/L NH_4_Cl, 0.75 g/L (NH_4_)_2_SO_4_, 1 g/L MgSO_4_·7H_2_O, 10.0 mg/L CaCl_2_·2H_2_O, 0.5 g/L ZnSO_4_·7H_2_O, 0.25 mg/L CuCl_2_·2H_2_O, 2.5 mg/L MnSO_4_·H_2_O, 1.75 mg/L CoCl_2_·6H_2_O, 0.12 mg/L H_3_BO_3_, 1.77 mg/L Al_2_(SO_4_)_3_, 0.5 mg/L Na_2_MoO_4_·2H_2_O, 16.1 mg/L ferric citrate, 20.0 mg/L thiamine, and 2.0 mg/L biotin). Sterilized glucose was supplemented separately into the medium to a final concentration of 30 g/L for seed culture. 0.1 mM isopropyl-beta-d-thiogalactopyranoside (IPTG) for the induction of genes expression was added when cells growth reached OD_600_ of 0.6–0.8. In addition, a certain amount of methanol and sodium formate were added, and cells were incubated for further 48 h at 37 °C, 200 rpm. When required, ampicillin was added to achieve the final concentrations of 100 μg/mL.

### Enzyme assays

The Mdh activity was measured as described previously (Whitaker et al. 2017). Briefly, cells were grown in M9 medium supplemented with 30 g/L glucose and induced with 0.1 mM IPTG when the OD_600_ was 0.6–0.8. Cells were collected and washed twice with 50 mM K_2_HPO_4_ buffer supplemented with 5 mM MgSO_4_. Cell pellets were re-suspended in M9 medium and adjusted to OD_600_ of 1.0. The experiments started after the addition of 0.2 M methanol. 800 μL sample was taken every 10 min, which were centrifuged for 2 min at 12,000 rpm at 4 °C to remove the cells. A total of 600 μL of the supernatant was mixed with 600 μL Nash reagent and the formaldehyde concentration was determined according to Nash (1981). Throughout the experiment, cultures were kept at 37 °C in a shaking water bath for 1 h. One unit (U) was defined as 1 μmol formaldehyde produced per min.

To determine the activity of Fdh, cells were harvested and washed as described above. Cells were suspended in 15 mL of ice cold 10 mM sodium phosphate buffer at pH 7.5 with 0.1 M β-mercaptoethanol and centrifuged for 8 min at 6000 rpm at 4 °C. The pellet was resuspended in 15 mL of ice cold 10 mM sodium phosphate buffer at pH 7.5 with 0.1 M β-mercaptoethanol and sonicated for 15 min in an ice bath using a 3 s interval between each cycle. After centrifuged, 200 μL of the supernatant was added to 2 mL containing 1.67 mM NAD^+^, 167 mM sodium formate, 100 mM β-mercaptoethanol in 10 mM phosphate buffer at pH 7.5 and kept at 30 °C (Zhang et al. 2018a). The absorbance of NADH at 340 nm was measured using a spectrophotometer. One unit (U) was defined as 1 μmol NADH produced per min.

The activity of Pyc was performed as described previously with some modifications. The cell extract was prepared, resuspended, and sonicated as described above. The enzymatic activity was measured according to the consumption of NADH at 340 nm in 1 mL Tris–HCl buffer (pH 7.4) containing 10 mM KHCO_3_, 10 mM MgCl_2_, 5 mM ATP, 0.1 mM NADH, 2 mM pyruvate, 200 μL cell extracts and 10 units of malate dehydrogenase.

### Anaerobic fermentation

Overnight cultures were inoculated to 500 mL flask containing 100 mL M9 medium with ampicillin antibiotics for aerobic growth at 37 °C and 200 rpm. After incubated for 10 h, 10% inoculum was transferred to 100 mL sealed anaerobic bottles containing 50 mL M9 medium supplemented with 30 g/L glucose, 0.1 mM IPTG and 6.4 g/L methanol. Adequate sodium formate was also added as required. Cells were grown at 37 °C and 200 rpm for 48 h under anaerobic condition. 16 g/L 4MgCO_3_·Mg(OH)_2_·5H_2_O was added to maintain the pH at neutral. The anaerobic condition was realized by the flush of oxygen-free CO_2_ for 4 min before the sterilization.

Bioreactor fermentations were performed in a 5-L bioreactor containing 2.0 L of CD medium with initial glucose concentration of 50 g/L. Fermentation was operated at 37 °C and 200 rpm. The pH was maintained to 6.8 by automatic addition of 20% Na_2_CO_3_. CO_2_ was flushed at a flow rate of 0.2 L/min to maintain anaerobic conditions. When cells grew to OD_600_ of 1.0, 0.1 mM IPTG was added. When the glucose concentration was lower than 10 g/L, 600 g/L glucose was fed into the fermentation medium to maintain the final concentration at 50 g/L. For C1-substrates co-feeding, 16 mL methanol (final concentration about 0.2 M) and 13 mL of 450 g/L sodium formate (final concentration is equivalent to 2 g/L formic acid) were added at 12 h. To maintain the concentration of sodium formate, 6.5 mL of 450 g/L sodium formate solution was added at 36 h, 48 h, and 60 h, respectively. The methanol evaporation rate along with any native methanol oxidation was determined from cultures of the empty vector of the control strain (G0) (Zhang et al. 2018a).

### Fed-batch with the intervention of polymer hollow fiber membrane

*Escherichia coli* cells were immobilized on the glycosylated membrane surface of polymer hollow fiber (PHF) membrane, which has been used to enhance succinic acid production in *A. succinate* 130Z in our pervious study (Gao et al. 2021). PHF membrane and glycosylated membrane were prepared by our previous study.

### Analytical methods

Biomass concentration was determined by measuring the OD_600_ using an AOE INSTRUMENTSUV1800 spectrophotometer. Methanol was measured via capillary gas chromatography (GC) using an Agilent 7890A gas chromatograph (Agilent Technologies, Waldbronn, Germany). Glucose concentration in the fermentation broth was detected by biosensor (Sieman, S-10). Organic acids were measured by high performance liquid chromatography (UitiMate 3000 HPLC system, Dionex, USA) equipped with a UVD 170U ultraviolet detector at a wavelength of 215 nm and an ion exchange column (Bio Rad Aminex HPX-87H column, USA). The mobile phase was 5 mM H_2_SO_4_ with a flow rate of 0.6 mL/min at 55 °C. The intracellular concentrations of NADH and NAD^+^ were assayed using a cycling method (Leonardo et al. 1996).

## Results and discussion

### Metabolic construction of methanol dissimilation module in *E. coli* Suc260

**Fig. 2:**
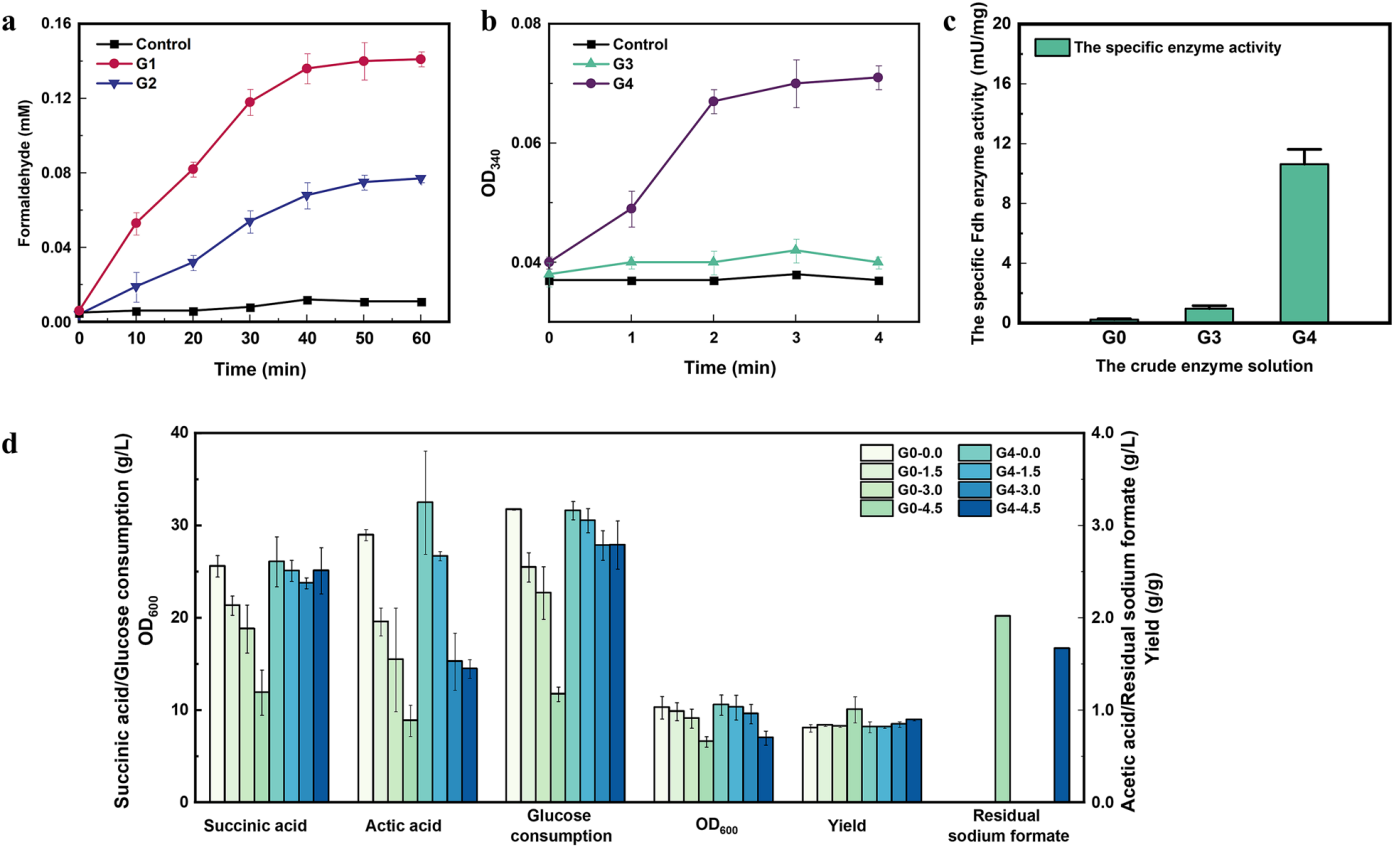
**a** Assessment of the two Mdhs by analyzing formaldehyde accumulation. **b** Assessment of the two Fdhs by analyzing NADH formation and **c** the calculated enzyme activities. **d** Optimization of sodium formate concentration gradient

To construct the methanol dissimilation pathway in the non-methylotrophic *E. coli*, a methanol dehydrogenase (Mdh) with high catalytic activity could be introduced. Mdh from *B. methanolicus* and *B. stearothermophilus* are two common candidates. The former one showed a high activity of 21.3 mU/mg in vivo (Zhang et al. 2018a), while the latter one showed a higher affinity towards methanol (Gonzalez et al. 2018; Whitaker et al. 2017). In this study, these two Mdhs (i.e., *Bm*Mdh and *Bs*Mdh) were ligated to the plasmid pTrc99A and expressed in *E. coli* Suc260, respectively, generating recombinant strains G1 and G2. Then, the enzymatic activities of *E. coli* cell suspensions were assayed. Compared to the control strain G0 with empty vector, formaldehyde concentrations in strains G1 and G2 were both increased when cells were exposed to methanol, indicating the vital function of Mdhs in the improvement of methanol assimilation (Fig. 2a). Within the two Mdhs, *BmMdh* showed higher activity than *Bs*Mdh in *E. coli* Suc260, reflected by a twofold higher formaldehyde concentration at 60 min (0.14 mM vs. 0.08 mM).

To further verify whether these enzymes are functionally expressed in *E. coli* under anaerobic conditions, the growth and fermentation performance of the recombinant strains were assessed. Consistent with the characterization of enzyme activity, the fermentation profiles using *BmMdh* also performed better than *Bs*Mdh when 30 g/L glucose and 200 mM methanol [the optimal concentration used in our previous study (Zhang et al. 2018a)] were co-fed in anaerobic bottles (Table 2). In details, strain G1 showed higher methanol consumption capability (0.22 g/L), resulting in the highest OD_600_ of 11.12. As a comparison, recombinant strain G2 consumed 0.15 g/L methanol, while the OD_600_ (9.44) was even lower than the control G0 (10.23). Owing to the additional provide of NADH through the methanol oxidation process, strain G1 produced 25.79 g/L succinic acid with a yield of 0.83 g/g, which was 5.6% and 2.5% higher than strain G0, respectively. Nevertheless, the succinic acid production in strain G2 was decreased with a lower titer (24.27 g/L) and yield (0.80 g/g). In terms of by-products accumulation, the introduction of Mdh enhanced formic acid accumulation both in stain G1 and G2 (0.65 g/L and 0.58 g/L vs. 0.53 g/L), which was due to the oxidation of methanol. The titer of acetic acid produced by strain G1 and G2 dropped to 2.65 g/L and 2.89 g/L, respectively, both lower than the control (3.12 g/L). Similar to the dissimilation of methanol, the accumulation of acetic acid is also accompanied by the regeneration of ATP (Li et al. 2016). In this case, the addition of methanol will relieve the pressure to provide more ATP through the production of acetic acid, resulting in the decrease in acetic acid accumulation. Taken together, compared with stain G2, stain G1 was superior in both enzyme activity and fermentation performance. Accordingly, strain G1 was chosen for following studies.

**Table 2:** Effects of introducing NAD^+^-dependent Mdhs on cell growth and fermentation performance in strain G0, G1, and G2

	Succinic acid (g/L)	Acetic acid (g/L)	Malic acid (g/L)	Formic acid (g/L)	Glucose consumption (g/L)	Methanol consumption (g/L)	Yield (g/g)	OD_600_
G0	24.42 ± 1.22	3.12 ± 0.26	0.25 ± 0.04	0.53 ± 0.11	30.18 ± 0.96	0.07 ± 0.01	0.82 ± 0.06	10.23 ± 1.03
G1	25.79 ± 0.77	2.65 ± 0.02	0.44 ± 0.03	0.65 ± 0.07	30.94 ± 1.07	0.22 ± 0.02	0.84 ± 0.05	11.12 ± 1.26
G2	24.27 ± 1.09	2.89 ± 0.19	1.30 ± 0.02	0.58 ± 0.25	30.35 ± 1.55	0.15 ± 0.01	0.80 ± 0.08	9.44 ± 0.08

### Identification of proper formate-converting enzymes in *E. coli* Suc260

In the methanol dissimilation pathway, formate can be converted to CO_2_ by formate dehydrogenase (Fdh) together with the regeneration of NADH. However, formate can also connect with tetrahydrofolate (H_4_F) to generate methylene-H_4_F without NADH generation (Jiang et al. 2021a). To increase the in vivo NADH pools, proper *Fdh* element can be introduced into strain G1, by which more formate could be directed to provide more NADH and improve the succinic acid yield. Previous studies have proved that Fdh from *C. boidinii* (*Cb*Fdh) and *M. extorquens* (*Me*Fdh) possessed high enzymatic activities when expressed in *E. coli* SBS550MG (Balzer et al. 2013) and *M. succiniciproducens* LPK7 (Ahn et al. 2017). Accordingly, *Me*Fdh and *Cb*Fdh were expressed in strain G1 in this study, resulted in stain G3 and G4, respectively. As shown in Fig. 2b and c, NADH was quickly regenerated from NAD^+^ in the reaction solution with *Cb*Fdh added, resulting in a rapid increase of OD_340_ from 0.040 to 0.067 within 2 min. By contrast, *Me*Fdh showed a lower catalytic activity, as OD_340_ was hardly increased within 4 min, indicating that almost no NADH was regenerated. The specific enzyme activity of the crude enzyme solution of strain G4 was 10.62 mU/mg, which was 11-fold higher than that of strain G3 (0.97 mU/mg). Obviously, *Cb*Fdh was supposed to possess a better capability for formate co-utilization.

As known, high concentration of formate is toxic to *E. coli*, while low concentration of formate cannot provide sufficient NADH. Therefore, it is essential to maintain an appropriate formate concentration to balance the cell growth and succinic acid production. Accordingly, a concentration gradient of sodium formate was chosen (0.0 g/L, 1.5 g/L, 3.0 g/L, 4.5 g/L, and 6.0 g/L, equivalenting to 0.0 g/L, 1.0 g/L, 2.0 g/L, 3.0 g/L, and 4.0 g/L formic acid, respectively). As shown in Fig. 2d, whether for strain G0 or strain G4, the addition of sodium formate showed inhibition to cell growth, as OD_600_ was decreased with the increase of sodium formate concentration. When sodium formate concentration reached 6.0 g/L, the cell growth was completely inhibited. Despite the growth inhibition, with the increase of sodium formate concentration, acetic acid accumulation was decreased, and succinic acid yield was improved. On the other hand, comparing the fermentation performance of strain G4 and G0, G4 showed higher succinic acid production, glucose consumption and higher OD_600_, indicating the contribution of Mdh and Fdh. Overall, higher formate concentration led to higher succinic acid yield, while caused severe growth inhibition. In this case, 3.0 g/L of sodium formate (2.0 g/L formic acid equivalent) might be the optimal concentration, as succinic acid yield was improved, and cell growth was not significantly affected.

### Fed-batch fermentation of strain G4 with co-feeding C1-substrates

**Fig. 3:**
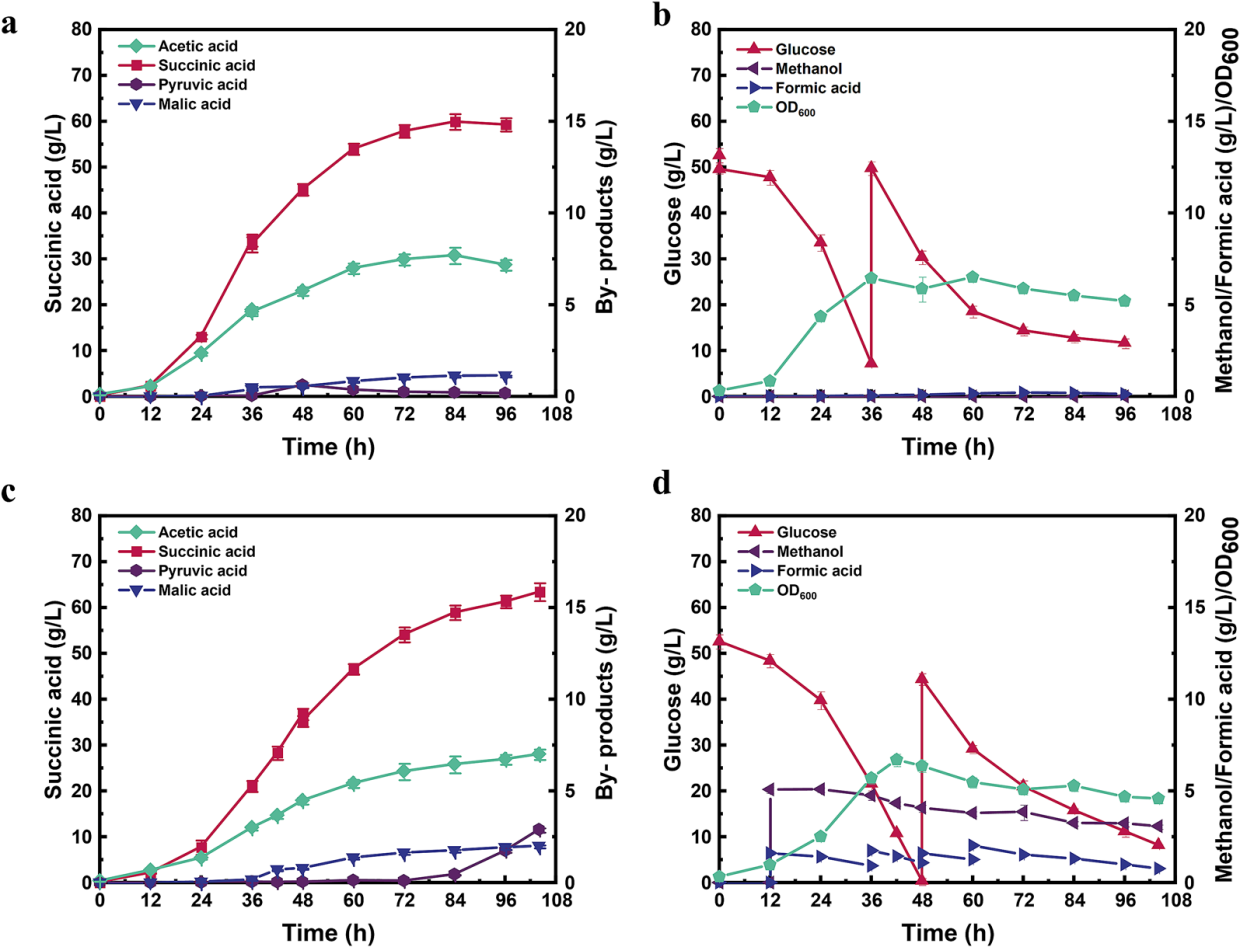
**a**, **b** Fed-batch fermentation of strain G4 with pure glucose feeding. **c**, **d** Fed-batch fermentation of strain G4 with methanol and sodium formate co-feeding

Fed-batch fermentation of strain G4 was performed in a 5-L bioreactor containing M9 medium with 50 g/L glucose, 200 mM methanol and 3.0 g/L sodium formate (2.0 g/L formic acid equivalent) as carbon sources. Due to the toxicity to cells, sodium formate was maintained below 2.0 g/L during the fermentation. The fed-batch fermentation of strain G4 without methanol and formate gave 58.72 g/L succinic acid from 83.50 g/L glucose with a yield of 0.91 g/g (to be noted, due to the increase in broth volume during the fermentation process, the glucose consumption used to calculate the yield is the total sugar mass divided by the final broth volume, which was lower than the fermentation profiles shown) after 96 h (Fig. 3a). Owing to the endowed ability to assimilate formic acid, no formic acid accumulation could be detected in strain G4 in this case (Fig. 3b). While the supplementation of methanol and formate could improve the succinic acid production to 63.42 g/L from 88.40 g/L glucose with a yield of 0.95 g/g, which was 8% higher than the control (Fig. 3c).

By-products accumulation was also increased when methanol and formate were co-fed. As shown in Fig. 3c, 2.91 g/L pyruvic acid was finally produced by strain G4 at 104 h. As a comparison, pyruvic acid accumulation was negligible during the fermentation in sole glucose culture (Fig. 3a). It appeared that succinic acid accumulation had reached a bottleneck at 84 h, leading partial flux from PEP to be allocated to pyruvic acid. This explanation could also be confirmed by the increased acetic acid accumulation. As seen in Fig. 3a and c, acetic acid accumulation with co-feeding of methanol and formate was lower than the control within 84 h (6.46 g/L vs. 7.70 g/L). However, after 84 h of fermentation, acetic acid accumulation kept increasing and finally reached 7.1 g/L, which is similar to the control. The accumulation of pyruvic acid and acetic acid was both increased significantly in the late fermentation period, which was probably caused by the feedback inhibition due to the large accumulation of succinic acid. Given that the production of pyruvic acid and acetic acid is accompanied by the generation of ATP, it appears to indicate the lack of intracellular energy in the late fermentation stage. It should also be noticed that malic acid was also increased by 70% with C1-substrates co-feeding (1.93 g/L vs. 1.14 g/L at 96 h). Since the conversion of malic acid to succinic acid requires NADH (Dai et al. 2020; Zhang et al. 2018a), the increased malic acid accumulation likely indicates that the intracellular NADH was still lacking, even if the oxidation of methanol and formate has provided additional NADH.

The insufficiency of intracellular ATP and NADH was not only reflected in the enhanced by-products generation, but also in the consumption of C1-substrates. To accurately detect methanol consumption, a blank control is set to evaluate the volatile loss of methanol, in which 1.02 g/L methanol was consumed. Therefore, accounting for the loss of the blank control, a total of 0.97 g/L methanol and 10.70 g/L sodium formate (equivalent to 7.13 g/L formic acid) was consumed by strain G4, indicating 0.24 mol/L NADH was provided. In contrast, only 0.38 g/L methanol consumption occurred by the recombinant *E. coli* Suc460 in our previous study, in which the methanol assimilation pathway was endowed (Zhang et al. 2018a). Different from the assimilation pathway that transforms C1-substrates into the central carbon metabolism, the dissimilation pathway is mainly responsible for providing energy and NADH through a series of oxidation reactions (Guo et al. 2021). Hence, the more C1-substrates consumption in this study may reflect the urgent needs for energy and NADH of cells. On the other hand, the consumption of methanol is much lower than that of formate (10.70 g/L vs. 0.97 g/L), which is likely due to the toxic effect of formaldehyde. As the methanol assimilation will generate formaldehyde first, its consumption rate must be restricted at a low level to avoid the overproduction of formaldehyde (Wang et al. 2020).

**Fig. 4:**
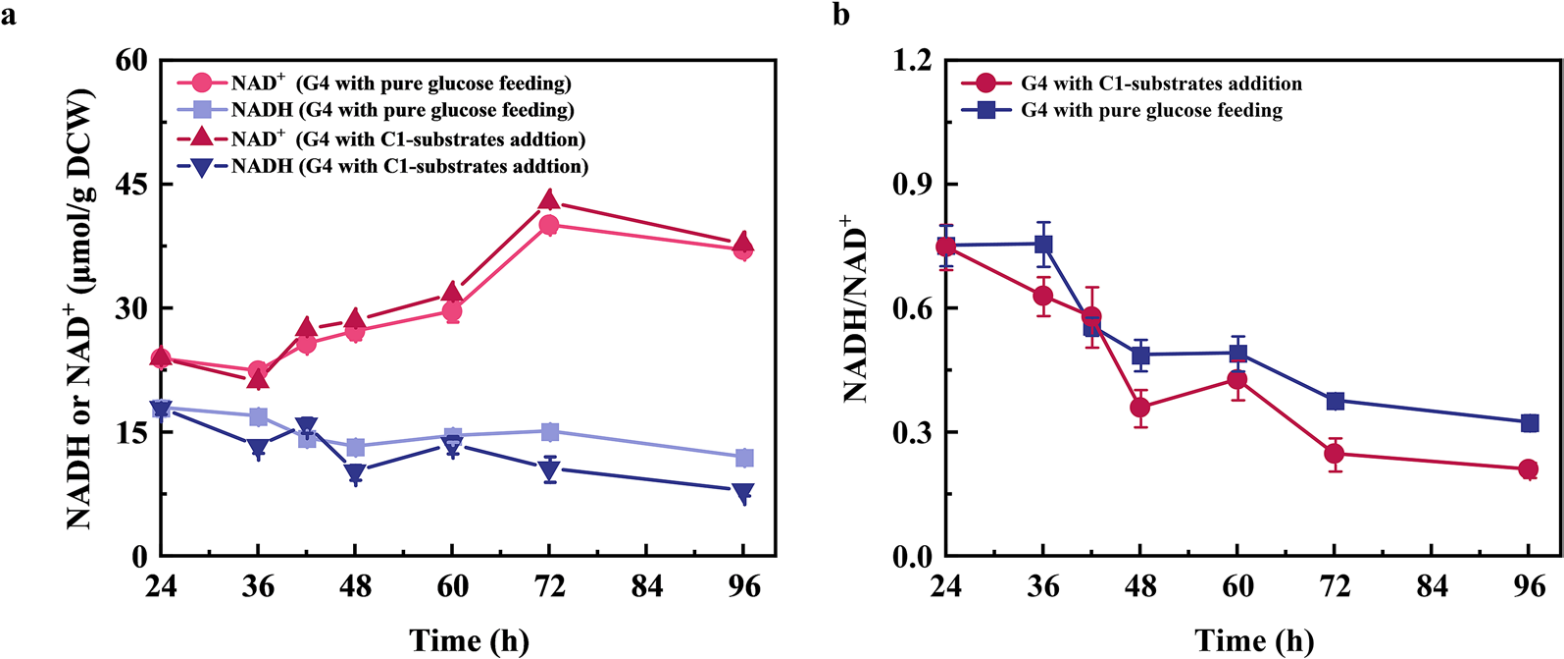
Time-course of the NADH, NAD^+^, and NADH/NAD^+^ ratio during fermentation. **a** NADH and NAD^+^ contents; **b** and NADH/NAD^+^ ratio

To evaluate the change of NAD(H) pools with the addition of methanol and formate, the total amount and ratio of NADH and NAD^+^ was assessed. As shown in Fig. 4, the NADH content was gradually decreased, while NAD^+^ content was increased accordingly no matter whether C1-substrates were supplemented or not. This might be due to that the succinic acid production continuously consumed NADH (Dai et al. 2020). After 96 h of fermentation, the NADH content with methanol and formate co-feeding was decreased from 17.92 to 7.94 μmol/g DCW, which was only 66% of the control with sole glucose (from 17.98 to 12.02 μmol/g DCW). Correspondingly, the ratio of NADH/NAD^+^ was gradually decreased from 0.75 to 0.21 when C1-substrates was co-fed, which was 34% lower than the control (from 0.75 to 0.32). This finding is also consistent with our pervious study, in which the addition of methanol resulted in a lower NADH level during the process of succinic acid production, implying that the NADH provided by C1-substrates oxidation is not sufficient to meet the demand for the increased succinic acid production (Zhang et al. 2018a). One possible explanation could be that the supplementation of methanol perturbs the cell membrane and affect the coupling efficiency of NADH and ATP regeneration (Eshinimaev et al. 2002; Zhang et al. 2018a). Previous studies have indicated that lower NADH/NAD^+^ ratio is an efficient strategy for succinate production (Li et al. 2013). Li et al. reduced NADH/NAD^+^ ratio by 24% by introducing fumarate reductase, resulting in a 13% increase in succinate yield (Li et al. 2017).

### Redirection of carbon dioxide for succinic acid production

**Fig. 5:**
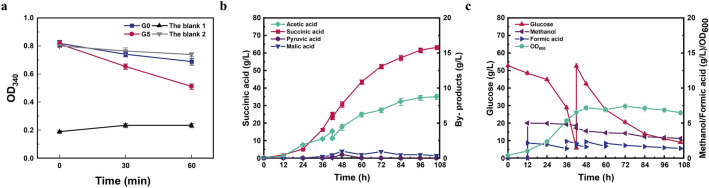
**a** Measuring the extracellular enzyme activity of Pyc in strain G5, the blank 1 and 2 were the reaction solution without NADH and pyruvic acid, respectively. **b**, **c** Fed-batch fermentation of strain G5 with C1-substrates addition

The above results have proved that the metabolically engineered strain G4 showed higher succinic acid production with methanol and formate as the auxiliary substrates. Nevertheless, pyruvic acid was surprisingly accumulation during anaerobic fermentation. Moreover, partial carbon sources entering the dissimilation pathway are still converted to CO_2_ and dissipated, although it could be converted to oxaloacetate by the catalysis of phosphoenolpyruvate carboxylase. To reduce the production of pyruvic acid and enhance the fixation of CO_2_, a heterologous pyruvate carboxylase from *L. lactis* (*Llpyc*) was further introduced into strain G4, resulting in strain G5, which can redirect pyruvate and CO_2_ into oxaloacetate (Fig. 1). *Llpyc* has been adopted to enhance succinic acid production in *E. coli* NZN111 or AFP111, as well as reducing pyruvic acid (Gokarn et al. 1998; Sanchez et al. 2005). To verify whether the heterologous *pyc* have been efficiently expressed in *E. coli*, the extracellular enzyme activity of *Llpyc* in strain G5 was first evaluated. As shown in Fig. 5a, the enzymatic activity of strain G5 crude solution showed a significant decrease in OD_340_ (from 0.84 to 0.51), indicating the consumption of NADH. As a comparison, OD_340_ of control strain G0 showed a lower decrease (from 0.82 to 0.69), which was similar to the blank control without the addition of crude enzyme solution (0.80 to 0.74), further implying the consumption of NADH in the recombinant strain G5.

Fed-batch fermentation was also performed using strain G5 under the similar fermentation conditions as strain G4 (Fig. 5b and c). As expected, the by-product of pyruvic acid was not detected in the fermentation broth using stain G5, indicating that the introduced *Llpyc* could successfully redirect pyruvate for oxaloacetate generation. However, only 63.16 g/L succinic acid was produced by recombinant strain G5 with the consumption of 90.52 g/L glucose, 1.12 g/L methanol and 9.89 g/L formate, equivalent to 0.25 mol/L NADH. The corresponding succinic acid yield was 0.94 g/g, which was slightly lower than that of strain G4 (0.95 g/g). Notably, 8.77 g/L acetic acid was accumulated using strain G5, which was 21.41% higher than that of strain G4. The overaccumulation of acetic acid indicated that higher energy requirement occurred in strain G5, as the generation of acetic acid is always coupled with the synthesis of ATP (Li et al. 2016). As known, the CO_2_ fixation process catalyzed by Pyc usually consumes ATP (Guo et al. 2021). Therefore, the introduction of *Llpyc* aggravated the energy burden of strain G5, thereby forcing it to provide additional energy through the generation of acetic acid, leading to the decrease of succinic acid production.

### Succinic acid production by glycosylated membrane-integrated biofilm

More ATP is required when cells resist adverse environments, such as high osmotic pressure and toxic substrates including methanol and formic acid. To further improve succinic acid production from methanol and formate, biofilm-based cell-immobilized fermentation could be a viable strategy. As known, biofilms are the structured microbial communities including fungi, bacteria, and algae etc., which help microorganisms adapt to different harsh micro-environmental conditions (pH, hyperosmosis, etc.) and tolerate toxic inhibitors (Gross et al. 2007; Jiang et al. 2021b). In our previous study, a specific glycosylated membrane has been designed to enhance the succinic acid production from glucose, which allowed cells to adhere on glycosylated PHF membrane and form biofilms of *A. succinogenes* 130Z (Gao et al. 2021). Therefore, this glycosylated membrane was adopted in this study to immobilize strain G5 and improve the final succinic acid production efficiency from glucose and C1-substrates.

**Fig. 6:**
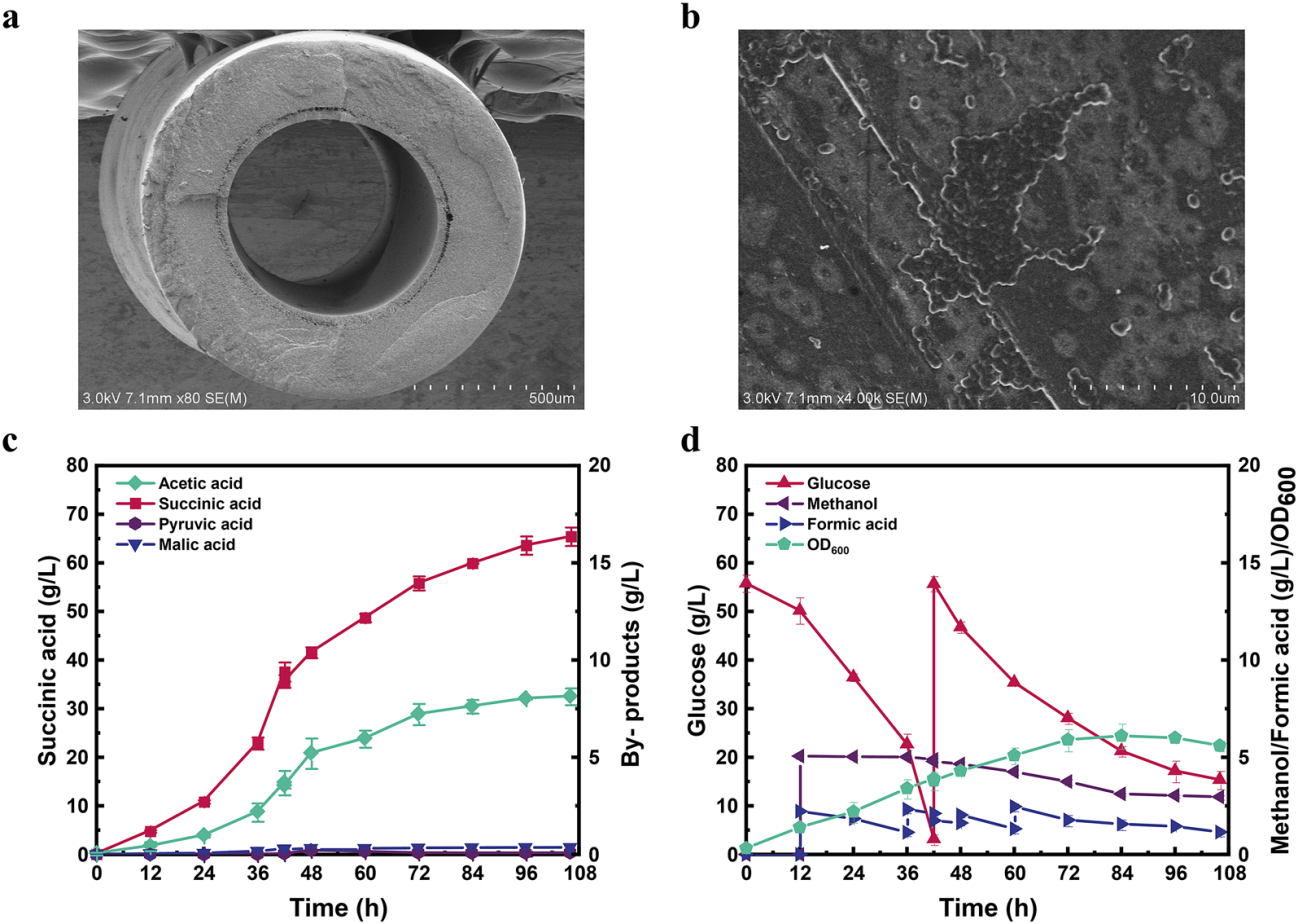
Immobilize cells with hollow fiber membrane PVDF. **a** SEM analysis of hollow fiber membrane morphology. **b** *E. coli* adhering on glycosylated PHF membrane with a large mount cell and forming biofilm. **c**, **d** Fed-batch fermentation of strain G5 with biofilm-based cell-immobilized fermentation

To verify whether the glycosylated membrane could immobilize *E. coli* cells, the membrane surface was first observed by scanning electron microscope (SEM). As showed in Fig. 6b, *E. coli* cells can adhere on the glycosylated membrane and form biofilm after 3 days. Fed-batch fermentation with PHF membrane intervening was further performed under the similar fermentation conditions. As seen in Fig. 6c and d, the increase of OD_600_ was lower than that of free cell fermentation due to the adsorption of *E. coli* cells on the membrane (6.1 vs. 7.4 at 106 h). However, the constant increase of OD_600_ occurred during the PHF membrane intervened fermentation process. Succinic acid production also showed a uniform increase throughout the fermentation process, which reached 65.44 g/L at 106 h with the consumption of 92.90 g/L glucose, 1.01 g/L methanol and 10.27 g/L formate. The succinic acid yield was also increased from 0.94 to 0.98 g/g, which was 8.9% higher than the original strain (Table 3). To the best of our knowledge, this yield (0.98 g/g) is the highest yield of succinic acid produced by a single-stage fermentation of *E. coli*. Higher titers and yields can be achieved with dual phase fermentation. For instance, overexpression of the PEP carboxylase in the engineered *E. coli* strain SD121 (Δ*pflB*, Δ*ldhA* and Δ*ptsG*) led to 116.2 g/L of succinic acid with a yield as high as 1.13 g/g of glucose within 75 h (Wang et al. 2011), while it requires additional time and carbon source for aerobic growth. On the other hand, the accumulation of byproduct acetic acid was reduced from 8.77 to 8.15 g/L, indicating the energy burden was somewhat alleviated. Besides, the accumulation of other byproducts including pyruvic acid and malic acid were negligible during the fermentation period. Taken together, these results indicated that the biofilm-based cell-immobilized fermentation provides a promising alternative to improve the biochemicals production efficiency from toxic substrates, such as C1 compounds.

**Table 3:** Comparison of fed-batch fermentation by strain G4 and G5

	Succinic acid (g/L)	Acetic acid (g/L)	Glucose consumption (g/L)	Methanol consumption (g/L)	Sodium formate consumption (g/L)	Yield^a^ (g/g)	OD_600_
G0^b^	60.82 ± 0.98	10.05 ± 0.44	82.42	–	–	0.90 ± 0.01	7.4 ± 0.3
G4^b^	58.72 ± 1.44	7.19 ± 0.29	83.50	–	–	0.91 ± 0.02	6.5 ± 0.2
G4^c^	63.42 ± 1.95	7.01 ± 0.28	88.40	0.97 ± 0.12	10.70 ± 0.04	0.95 ± 0.03	6.8 ± 0.3
G5^c^	63.16 ± 1.15	8.77 ± 0.35	90.52	1.12 ± 0.10	9.89 ± 0.14	0.94 ± 0.02	7.4 ± 0.4
G5^c,d^	65.44 ± 1.90	8.15 ± 0.43	92.90	1.01 ± 0.11	10.27 ± 0.18	0.98 ± 0.03	6.1 ± 0.6

^a^Due to the volume expansion in fed-batch fermentation, the measured glucose consumption in real time could not reflect the true yield. Real glucose consumption was calculated by dividing the total added glucose by the final total volume, which would be lower than the measured value

^b^Fermentation with pure glucose feeding

^c^Fermentation with methanol and sodium formate co-feeding

^d^Biofilm-based cell-immobilized fermentation

## Conclusions

The oxidation of C1 substrates can release NADH, which has been proved to increase the yield of reduced products, such as succinic acid. In this study, methanol dissimilation pathway was first introduced into a succinic acid producer *E. coli* Suc260 by employing Mdh2 from *B. methanolicus* MGA3 and Fdh1 from *C. boidinii*. The resulting strain G4 produced 63.42 g/L succinic acid in fed-batch fermentation with a yield of 0.95 g/g. Furthermore, Pyc from *L. lactis* was introduced into strain G4 to eliminate by-product pyruvic acid and fix CO_2_ to succinic acid. The resulting strain G5 did not accumulate pyruvic acid, while succinic acid yield was dropped to 0.94 g/g, which may be due to the insufficient ATP supply. To this end, a glycosylated membrane was adopted to immobilize *E. coli* in the fed-batch, aiming to relieving the energy stress on cells. Finally, 65.44 g/L succinic acid was obtained and the yield was improved to 0.98 g/g. It is generally known that NADH/NAD^+^ play a major role in the production of different fermentation products. Several previous studies have tried to increase intracellular NADH by introducing formate dehydrogenase and have demonstrated its role in increasing reduced metabolites concentration including succinate, ethanol, and lactate (Berrios-Rivera et al. 2002a, b). This study proved that endowing producers with methanol dissimilation pathway to provide additional NADH could effectively improve succinic acid production, which would be of great value for biological conversion of methanol to other reductive products.

### Supplementary Information

**Additional file 1: Table S1.** Sequences of primer pairs used in this study.

## Data Availability

All data supporting this article’s conclusion are available.
