# Supplementary material for: Improved succinic acid production through the reconstruction of methanol dissimilation in Escherichia coli
Source: Bioresour Bioprocess. 2022 May 31;9(1):62. doi: 10.1186/s40643-022-00547-x (PMC10991533; doi:10.1186/s40643-022-00547-x)
Supplement: Supplementary file 1 — Additional file 1: Table S1. Sequences of primer pairs used in this study. [file 40643_2022_547_MOESM1_ESM.docx]

Additional file 1

**Improved succinic acid production through the reconstruction of methanol dissimilation in *Escherichia coli***

Feng Guo^a^, Min Wu^a^, Shangjie Zhang^a^, Yifan Feng^a^, Yujia Jiang^a^,

Wankui Jiang^a^, Fengxue Xin^a,b*^, Wenming Zhang^a,b*^, Min Jiang^a,b^

^a^ State Key Laboratory of Materials-Oriented Chemical Engineering,

College of Biotechnology and Pharmaceutical Engineering,

Nanjing Tech University, Nanjing, 211800, P.R. China

^b^ Jiangsu National Synergetic Innovation Center for Advanced Materials (SICAM), Nanjing Tech University, Nanjing, 211800, P.R. China

^*^ Corresponding authors at:

State Key Laboratory of Materials-Oriented Chemical Engineering, College of Biotechnology and Pharmaceutical Engineering, Nanjing Tech University, Puzhu South Road 30#, Nanjing 211800, P. R. China.

E-mail addresses: [zhangwm@njtech.edu.cn](mailto:zhangwm@njtech.edu.cn) (W.M. Zhang); [xinfengxue@njtech.edu.cn](mailto:xinfengxue@njtech.edu.cn); (F.X. Xin)

# Table S1 Sequences of primer pairs used in this study

| Primers | Sequences |
| --- | --- |
| mdh1-F | aggaaacagaccatggAATTCATGACCAATACCCAGAGC |
| mdh1-R | ccgggtaccgagctcgAATTCTTACATGGCATTTTTGATAATC |
| mdh2-F | aggaaacagaccatggAATTCgttgacaattaatcatccggctc |
| mdh2-R | ccgggtaccgagctcgAATTCTTAATCCTCTTTCAGCTTCAGCA |
| fdh1-F | ggtacccggggatccTCTAGAATGAAGATCGTTCTGGTGCTG |
| fdh1-R | tgcctgcaggtcgacTCTAGATTATTTCTTATCGTGTTTGC |
| fdhABCD-F | GGTACCCGGGGATCCTCTAGAATGGTCAGTCCCGAGCCTTG |
| fdhABCD-R | TGCCTGCAGGTCGACTCTAGATTACCCCGCGTCGCTGG |
| fdh1-trc-F | ggtacccggggatccTCTAGAttgacaattaatcatccggctc |
| fdh1-trc-R | TGCCTGCAGGTCGACTCTAGATTATTTCTTATCGTGTTTGC |
| fdhABCD-trc-F | ggtacccggggatccTCTAGAttgacaattaatcatccggctc |
| fdhABCD-trc-R | TGCCTGCAGGTCGACTCTAGA TTACCCCGCGTCGCTGG |
| pyc-F | cggggatcctctagaATGAAAAAACTACTCGTCGCCAATC |
| pyc-R | gcaggtcgactctagaAGCTCTGGTAGGGTTAATTTAAAG |
| pyc-trc-F | ctgcaggcatgcaagcttttgacaattaatcatccggctcg |
| pyc-trc-R | caaaacagccaagcttAGCTCTGGTAGGGTTAATTTAAAG |
